# Protease Selection Influences Molecular Weight, In Vitro Antioxidant Activity and LO2 Cellular Protective Effects of Oyster Protein Hydrolysates

**DOI:** 10.3390/foods15061030

**Published:** 2026-03-16

**Authors:** Can Huang, Lu Li, Ruifang Wang, Guohong Wu, Hejian Xiong, Ying Ma

**Affiliations:** 1 College of Ocean Food and Biological Engineering, Jimei University, Xiamen 361021, China; 15256615287@163.com (C.H.); lllucy0606@163.com (L.L.); 200661000052@jmu.edu.cn (R.W.); 2 Daye Public Inspection and Testing Center, Huangshi 435100, China; 3 Fisheries College, Jimei University, Xiamen 361021, China; maying@jmu.edu.cn

**Keywords:** oyster peptide, molecular weight, DPPH radical scavenging, linoleic acid autoxidation inhibition, cellular antioxidant activity

## Abstract

This study compared the effectiveness of alkaline protease, neutral protease, trypsin, and papain in hydrolyzing oyster proteins and evaluated the antioxidant activities of the resulting hydrolysates. Alkaline protease achieved the highest degree of hydrolysis (30.96%) and the highest proportion of peptides ≤1 kDa (64.23%). Papain showed the lowest hydrolysis degree (18.29%). After separation by Sephadex G-15 gel filtration chromatography, the resulting low-molecular-weight peptide fractions (≤1 kDa) from each hydrolysate exhibited higher in vitro antioxidant activity than the higher-molecular-weight fractions (>1 kDa). Notably, trypsin and papain-derived low-molecular-weight fractions (OPP-T2 and OPP-P2) demonstrated stronger DPPH radical scavenging and inhibition of linoleic acid autoxidation than those from alkaline and neutral proteases. Cell experiments revealed that all low-molecular-weight fractions effectively alleviated H_2_O_2_-induced oxidative damage in LO2 cells. OPP-T2 and OPP-P2 exhibited significantly stronger protection of cell membrane integrity and enhancement of superoxide dismutase (SOD) activity than OPP-A2 and OPP-N2 (*p* < 0.05). OPP-T2 also showed the most pronounced increase in glutathione peroxidase (GSH-Px) activity (*p* < 0.05). These findings demonstrate that protease selection critically influences hydrolysis efficiency and antioxidant activity, with molecular weight being a key determinant of peptide antioxidant capacity. This work provides a reference for the development and application of oyster peptides.

## 1. Introduction

Excessive reactive oxygen species (ROS) generation disrupts redox homeostasis, impairing cellular antioxidant defenses and promoting oxidative injury to lipids and proteins. It is a significant contributor to aging, inflammation, metabolic syndrome, and other diseases [1,2,3]. Antioxidants are effective means to mitigate oxidative stress. Antioxidant peptides, as biological antioxidants, have garnered considerable attention due to their high bioactivity and good safety profile [4].

Natural resources of antioxidant peptides are relatively limited. Biocatalytic hydrolysis of protein resources is the most effective and environmentally friendly route to obtain antioxidant peptides [5]. However, the specificity of protease cleavage sites influences the structure and activity of the resulting peptides, making the choice of protease particularly important [6]. Hu et al. [7] and Liu J et al. [8] reported that papain- and trypsin-derived hydrolysates of abalone visceral proteins yielded small peptides such as TIDCDR and YHGF that exhibited strong radical-scavenging capacity and cellular antioxidant activity. Liu B et al. [9] demonstrated that peptides obtained through alkaline protease hydrolysis could reduce inflammatory cytokines including IL-1β, IL-6, and TNF-α while enhancing antioxidant enzyme activities such as SOD and GSH-Px and reducing malondialdehyde (MDA) levels. In addition, Zhang L et al. [10] showed that hydrolysates from alkaline protease hydrolysis of soy protein exhibited significant DPPH radical scavenging, metal chelating activity, and oxygen radical absorbance capacity. Mahmoud et al. [11] utilized papain-hydrolyzed soybean antioxidative peptides to develop functional biscuit products. However, current research on the preparation of antioxidant peptides through protein hydrolysis is often limited to optimizing the reaction conditions of a single enzyme. Comparative investigations examining how different proteases influence hydrolysis efficiency, peptide molecular weight distribution, and the resulting antioxidant activities of hydrolysates derived from the same protein source remain limited. Such a comparison forms the foundation for the rational selection of proteases in the production of high-value-added oyster protein hydrolysates for use in functional foods.

Marine organisms are rich in high-quality protein. Peptides generated from their enzymatic hydrolysis, with low antigenicity and high bioactivity, have become highly promising raw materials for active peptide development [12,13]. Oyster, as an important economic shellfish in China with abundant resources but traditionally low value-added processed products, is an ideal raw material for developing bioactive peptides [14,15]. In the present study, four proteases with different catalytic characteristics were used: alcalase (a broad-spectrum endopeptidase), neutrase and trypsin (which specifically target lysine and arginine residues), and papain (a cysteine endopeptidase with broad specificity). It was hypothesized that the distinct cleavage patterns of these enzymes would generate oyster protein hydrolysates with different molecular weight distributions and peptide sequences, thereby leading to significant differences in their antioxidant properties. To test this hypothesis, this study established the following objectives: first, to compare the hydrolysis efficiency and size distribution of oyster protein hydrolysates obtained with the four proteases; second, to fractionate the hydrolysates on a Sephadex G-15 column and evaluate their in vitro antioxidant activities; and third, to assess the protective effects of low-molecular-weight fractions against H_2_O_2_-induced oxidative injury in LO2 cells by analyzing cell viability, LDH activity, MDA content, and antioxidant enzyme activities. The aim was to investigate the influence of protease specificity on the antioxidant activity of oyster peptides, thereby providing a reference for the development and application of oyster antioxidant peptides.

## 2. Materials and Methods

### 2.1. Materials

Oysters were obtained from Dongshan Island, Fujian Province, China. After shelling and cleaning, they were portioned and stored at −20 °C. Trypsin and formic acid were obtained from Amresco Co., Ltd. (Cleveland, OH, USA). Alkaline protease and neutral protease were supplied by Novozymes Co., Ltd. (Tianjin, China). Papain, acetonitrile, trifluoroacetic acid (TFA), linoleic acid, 1,1-diphenyl-2-picrylhydrazyl (DPPH), and ammonium thiocyanate were obtained from Sigma-Aldrich Co., Ltd. (Burlington, NJ, USA). Sephadex G-15 gel was purchased from GE Healthcare Co., Ltd. (Chicago, Sweden). The bicinchoninic acid (BCA) protein assay kit, as well as kits for superoxide dismutase (SOD), glutathione peroxidase (GSH-Px), lactate dehydrogenase (LDH), malondialdehyde (MDA), and catalase (CAT), was sourced from Nanjing Jiancheng Bioengineering Institute Co., Ltd. (Nanjing, China). Ethanol and hydrogen peroxide were obtained from Sinopharm Chemical Reagent Co., Ltd. (Shanghai, China). The following reagents were procured from HyClone Co., Ltd. (Logan, UT, USA): penicillin–streptomycin solution, high-glucose Dulbecco’s modified Eagle’s medium (high-glucose DMEM), phosphate-buffered saline (PBS), 0.25% trypsin-EDTA, and thiazolyl blue tetrazolium bromide (MTT). All reagents used were of analytical grade unless otherwise specified.

### 2.2. Preparation of Oyster Hydrolysates

Following the method of Chen et al. [16], minced oyster meat was mixed with distilled water, homogenized, and heated in a water bath at 95 °C for 10 min, then crushed using a tissue homogenizer. The hydrolysis conditions for each enzyme were optimized using one-variable-at-a-time experiments and response surface methodology (RSM), and the detailed parameters are summarized in Table 1. The enzymatic reactions were allowed to proceed for 3 h under their respective optimal conditions. The detailed RSM data are provided in the Supplementary Materials (Tables S1–S5). After hydrolysis, enzymes were inactivated by heating the reaction mixtures in boiling water for 10 min. The mixture was then centrifuged at 4500× *g* for 10 min, and the obtained supernatants were collected, and stored as oyster hydrolysate powder.

### 2.3. Determination of Oyster Protein Hydrolysis Degree

The degree of hydrolysis (DH) was quantified via amino nitrogen content using formaldehyde titration. An aliquot of the hydrolysate (5 mL) was first diluted with deionized water to a final volume of 100 mL. From this, 20 mL was dispensed into a beaker containing 60 mL of deionized water, and the mixture was homogenized on a magnetic stirrer prior to pH electrode insertion. With continuous stirring, the pH was monitored as the sample was titrated with 0.05 M NaOH to an initial endpoint of 8.2. After adding 10 mL of 36% formaldehyde and mixing, titration resumed until pH 9.2. The volume of titrant consumed between pH 8.2 and 9.2 was recorded and used to calculate amino nitrogen content and DH, as per the following Formulas (1) and (2):

(1) $$\mathrm{Amino}\;\mathrm{nitrogen}\;\mathrm{content}\;(\%)\;=\;\left[C\;\times \;(V_{1}\;-{\;V}_{2})\;\times \;14\right]/5\;\times \;100$$

V_1_ and V_2_ denote the volumes (mL) of NaOH solution consumed by the sample and blank (prepared with deionized water), respectively; C is the molar concentration of NaOH (mol/L); and 14 is the millimolar mass of nitrogen (mg/mmol).

(2) Degree of hydrolysis (%)=m/M × 100

where m and M represent the amino nitrogen content (mg) in the hydrolysate and the total nitrogen content (mg) in the sample, respectively.

### 2.4. Determination of the Molecular Weight Distribution of Oyster Peptides

Gel permeation chromatography (GPC) [17] was employed to characterize the molecular weight distribution of the peptides, using a TSKgel G2000SWXL column (Φ 7.8 × 300 mm). The mobile phase, composed of acetonitrile, ultrapure water, and TFA (45:55:0.1, *v*/*v*/*v*), was pumped at a flow rate of 0.5 mL/min. Detection wavelength: 220 nm; Column temperature: 30 °C. Standards for calibration: Cytochrome C (MW 12,384.00 Da), Insulin (MW 5777.54 Da), Bacitracin (MW 1422.69 Da), Oxidized Glutathione (MW 612.64 Da), Gly-Gly-Gly (MW 189.17 Da).

### 2.5. Separation and Purification of Oyster Hydrolysates

#### 2.5.1. Gel Filtration Chromatography

Peptide fractions were separated using a Sephadex G-15 gel filtration column. Elution conditions: Sample concentration 50 mg/mL; Sample loading volume 2 mL; Ultrapure water as mobile phase; Flow rate 24 mL/h; Fractions collected every 10 min; Absorbance monitored at 220 nm. Eluates under the same peak were pooled and freeze-dried.

#### 2.5.2. Determination of Protein and Polysaccharide Contents

Protein content in the fractions was quantified by the Coomassie Brilliant Blue method; polysaccharides were similarly analyzed using the phenol–sulfuric acid assay, in accordance with established protocols [18].

### 2.6. Determination of in Vitro Antioxidant Activity of Oyster Peptides

#### 2.6.1. DPPH Radical Scavenging Activity

An ethanolic DPPH solution (0.2 mmol/L) was prepared fresh. Sample dilutions ranging from 1 to 10 mg/mL were made. The assay mixture consisted of 2 mL of sample solution and 2 mL of DPPH solution, which was incubated in darkness for 30 min at ambient temperature before measuring absorbance at 517 nm (denoted as A_0_). Two reference solutions were prepared under identical conditions: one substituting DPPH with absolute ethanol (to obtain A_1_) and another in which the sample solution was replaced with the solvent (absolute ethanol) (to obtain A_2_). The scavenging rate was computed according to Formula (3):

(3) $$\mathrm{DPPH}\;\mathrm{radical}\;\mathrm{scavenging}\;\mathrm{rate}\;(\%)\;=\;\left[1\;-\;(A_{0}\;-{\;A}_{1})/A_{2}\right]\;\times \;100$$

#### 2.6.2. Inhibition of Linoleic Acid Autoxidation

The inhibitory effect of the peptides on linoleic acid oxidation was evaluated following a previously reported method [19] with slight modifications. Peptide samples were prepared in 0.1 M phosphate buffer (pH 7.0) at different concentrations. The reaction mixture contained sample solution, linoleic acid solution, and deionized water, and the system was incubated at 60 °C under light-protected conditions. The peroxide value generated during oxidation was determined using the thiocyanate method. Absorbance was recorded at 500 nm, absorbance on day 0 was recorded as A (sample) and A_0_ (blank control). Absorbance after 7 days was recorded as A_1_ (sample) and A_2_ (blank control). The inhibition rate of lipid oxidation was calculated according to Formula (4):

(4) $$\mathrm{Inhibition}\;\mathrm{rate}\;(\%)\;=\;\left[\left(A_{1\;}-\;A)/(A_{0}\;-\;A_{1}\right)\right]\;\times \;100$$

### 2.7. H_2_O_2_-Induced Oxidative Damage in LO2 Cell

To establish an oxidative stress model, LO2 cells (obtained from the Cell Bank of the Chinese Academy of Sciences (Shanghai, China)) were first cultured in plates for 24 h to achieve adherence, followed by exposure to H_2_O_2_, as described in [20]. After washing with PBS, cells were exposed to H_2_O_2_ solutions at different concentrations (0–10 mmol/L) for 2 h to induce oxidative stress. Cell viability was subsequently determined using the MTT assay.

### 2.8. MTT Experiments

LO2 cells at logarithmic phase were plated in 96-well plates at a seeding density of 1 × 10^5^ cells/mL (100 μL/well) and cultured for 24 h to permit attachment (37 °C, 5% CO_2_). Following medium aspiration and PBS wash, the cells were treated with graded concentrations of oyster peptide samples (0.01–2 mg/mL), alongside control and blank groups. After 24 h of exposure, each well received 20 μL of MTT solution and was incubated for 4 h. The supernatant was then discarded, and 150 μL of DMSO was added to dissolve the formazan crystals. Absorbance was recorded at 490 nm using a microplate reader, and cell viability was derived from Formula (5):

(5) $$\mathrm{Cell}\;\mathrm{viability}\;(\%)\;=\;\left[(B\;-{\;A}_{0})/(A\;-{\;A}_{0})\right]\;\times \;100$$

where A, A_0_, and B represent the absorbance of the control, sample blank, and sample, respectively (*n* = 6).

### 2.9. Determination of MDA Content, and LDH, SOD, GSH-Px, and CAT Activities

Cells in the logarithmic phase were seeded in 6-well plates and incubated for 24 h. After treatment with peptide fractions, oxidative damage was induced using 2 mmol/L H_2_O_2_ for 2 h. The culture supernatant was collected to determine LDH activity. Cells were subsequently harvested, lysed by ultrasonic disruption, and centrifuged to obtain the supernatant. MDA content and the enzymatic activities of SOD, CAT, and GSH-Px were measured with commercially available kits as per the supplier’s guidelines.

### 2.10. Data Processing

All experimental data, with replicate numbers (*n*) specified in the respective table/figure legends, were initially processed in Microsoft Excel 2010 (Microsoft Corporation, Redmond, WA, USA) and presented as means ± SD. Statistical evaluation was conducted using SPSS Statistics 19.0 (IBM, Armonk, NY, USA). After confirming normality and homogeneity of variance, one-way ANOVA was applied to compare multiple groups, followed by Duncan’s multiple range test where significant differences existed. Significance was accepted at *p* < 0.05. Graphs were plotted with GraphPad Prism 9 (GraphPad Software, San Diego, CA, USA).

## 3. Results and Discussion

### 3.1. Hydrolysis Effects of Different Proteases on Oyster Protein

Proteases cleave peptide bonds, breaking down large proteins/peptides into smaller peptides. Different proteases exhibit varying degrees of hydrolysis due to differences in cleavage site specificity [20]. The hydrolysis effects of the four proteases are shown in Table 2. Alkaline protease achieved the highest degree of hydrolysis and produced the highest proportion of peptides ≤1 kDa. Similar observations have been reported in the enzymatic hydrolysis of quinoa protein by Gan et al. [21], who found that alcalase resulted in the highest hydrolysis efficiency among several tested enzymes and produced a peptide fraction (QPAH) ≤1 kDa accounting for 83% of the ultrafiltered hydrolysate. Alkaline protease is a broad-specificity endopeptidase; it preferentially cleaves peptide bonds located at the carboxyl side of hydrophobic or aromatic amino acid residues [22,23], which are abundant in oyster protein [24,25]. The alkaline environment hydrolysis favors protein unfolding, thereby contributing to efficient hydrolysis [26].

The degree of hydrolysis for trypsin was comparable to that of neutral protease but remained significantly lower than that achieved with alkaline protease (*p* < 0.05). Trypsin exhibits high substrate specificity and predominantly cleaves peptide bonds on the carboxyl side of lysine and arginine residues [27], resulting in the highest proportion of peptides >1 kDa. Papain showed the lowest degree of hydrolysis (*p* < 0.05), possibly due to its high sensitivity to environmental conditions. Disturbances in the microenvironment near its active site can alter the spatial conformation of the catalytic center, reducing activity [28,29]. Similar results have been observed in the hydrolysis of proteins from plants [30,31] and marine sources [32]. Zhang S et al. [33] used papain, pepsin, alcalase, trypsin, and neutrase to hydrolyze the muscle of skipjack tuna and found that the hydrolysis products of alkaline protease exhibited high angiotensin-I converting enzyme (ACE) inhibitory activity.

### 3.2. Separation and Purification of Oyster Hydrolysates

Dextran gel permeation chromatography separates enzymatically hydrolyzed active peptide fragments based on molecular weight differences, allowing for further purification of polypeptide components within specific molecular weight ranges. The oyster hydrolysates from the four enzymes were separated via Sephadex G-15 gel, yielding four large peptide fractions (OPP-A1, OPP-N1, OPP-T1 and OPP-P1) and four low-molecular-weight fractions (OPP-A2, OPP-N2, OPP-T2 and OPP-P2). Their compositions are shown in Table 3. High-molecular-weight peptide fractions (>1 kDa) also contained substantial polysaccharides, related to the high glycogen and glycoproteins content in oysters [34]. Low-molecular-weight fractions consisted mainly of peptides ≤1 kDa, with significantly higher protein and lower polysaccharide content due to more thorough hydrolysis.

### 3.3. Evaluation of in Vitro Antioxidant Activity of Oyster Peptides

**Figure 1:**
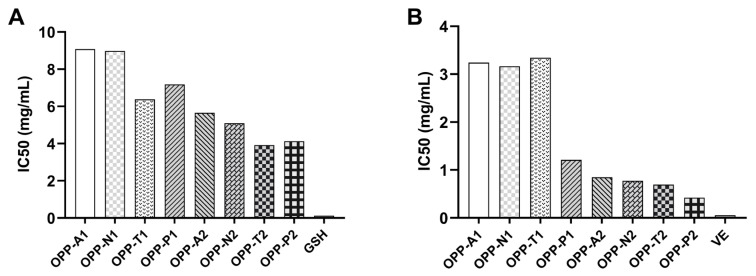
In vitro antioxidant activity of oyster peptides. (**A**) DPPH radical scavenging activity; (**B**) inhibition of linoleic acid autoxidation.

DPPH radical scavenging and linoleic acid autoxidation inhibition were used to evaluate in vitro antioxidant activity (Figure 1). In both assays, all low-molecular-weight fractions (OPP-A2, N2, T2, P2) showed higher activity than their corresponding large peptide fractions derived from the same enzymatic hydrolysates (OPP-A1, N1, T1, P1). Furthermore, all low-molecular-weight fractions were more active than all large peptide fractions, indicating that molecular weight is a key factor influencing the in vitro antioxidant activity of oyster peptides. This aligns with findings reported previously. For instance, Fazhi et al. [35] observed that tripeptides from fermented sesame meal exhibited significantly higher DPPH scavenging activity than tetrapeptides and hexapeptides. Similarly, Tian et al. [36] reported that wheat germ peptides with molecular weight < 3 kDa showed a DPPH scavenging rate of 85.29%, markedly superior to those >3 kDa. The enhanced activity of smaller peptides may be attributed to two factors. Peptides ≤ 1 kDa generally contain higher proportions of hydrophobic amino acids, facilitating interactions within lipid environments and improving antioxidant performance in lipid peroxidation systems [37]. Additionally, low-molecular-weight hydrolysates can efficiently donate protons to free radicals and stabilize reactive oxygen species through direct electron transfer due to their structural characteristics [38].

Beyond the molecular weight effect, significant differences existed among hydrolysates from different proteases. Trypsin and papain hydrolysates showed stronger DPPH scavenging than those from microbial alkaline and neutral proteases. OPP-P2 and OPP-T2 had IC_50_ values of 4.13 mg/mL and 3.92 mg/mL, respectively, which were lower than OPP-A2 (5.66 mg/mL) and OPP-N2 (5.10 mg/mL). Papain hydrolysates (OPP-P1, P2) exhibited the strongest inhibition of linoleic acid autoxidation, showing excellent potential against lipid peroxidation. Similar protease-specific activity trends have been reported. For instance, trypsin was identified as the most suitable enzyme for generating edible bird’s nest protein-derived antioxidant peptides [39], whereas papain-derived peptides from moth bean protein demonstrated superior ABTS scavenging and Fe^2+^ reducing activities compared to other enzymatic hydrolysates [40]. These functional variations among hydrolysates are likely attributable to the distinct cleavage specificities of the proteases employed, which generate peptides with differing amino acid sequences, hydrophobicity, and other structural features—even within the same molecular weight range—consequently leading to differential antioxidant performance [41].

Considering the superior antioxidant performance of the low-molecular-weight fractions, subsequent cellular experiments focused primarily on these peptide fractions.

### 3.4. Effect of Oyster Peptides and H_2_O_2_ on LO2 Cell Viability

**Figure 2:**
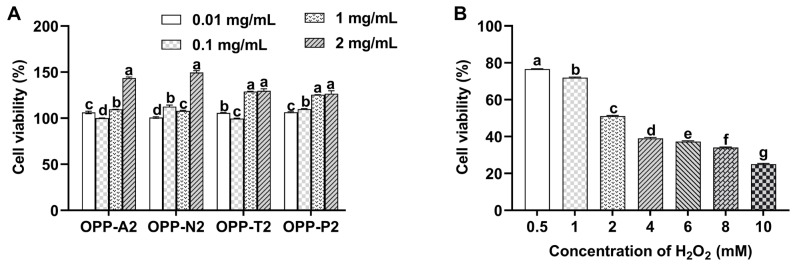
Effects of oyster peptides and H_2_O_2_ on LO2 cell viability. (**A**) Cell viability after 24 h treatment with oyster peptides at different concentrations. Bars annotated with distinct lowercase letters differ significantly within each peptide group (*p* < 0.05); (**B**) viability of cells treated with varying H_2_O_2_ concentrations for 2 h. Values represent means ± SD (*n* = 6). Different lowercase letters indicate statistical significance among H_2_O_2_ concentrations (*p* < 0.05).

As shown in Figure 2A, the four oyster peptide samples at tested concentrations (0.01, 0.1, 1, 2 mg/mL) promoted LO2 cell growth. Cell viability increased gradually with concentration, ranging from 95% to 150%. These concentrations were therefore used in subsequent experiments to evaluate protective effects.

H_2_O_2_ is a well-established inducer of oxidative damage, with concentration-dependent cytotoxic effects. The viability of LO2 cells following 2 h exposure to various concentrations of H_2_O_2_ is presented in Figure 2B. Cell viability decreased significantly with increasing H_2_O_2_ concentration (*p* < 0.05). Exposure to 2 mmol/L H_2_O_2_ reduced viability to 51.10%, approximating the half-lethal dose (LD_50_). A further increase to 4 mmol/L H_2_O_2_ lowered viability to 39.01%. Based on these results, 2 mmol/L H_2_O_2_ was selected to establish the oxidative injury model in LO2 cells.

### 3.5. Protective Effect of Oyster Peptides Against H_2_O_2_-Induced LO2 Cell Injury

**Figure 3:**
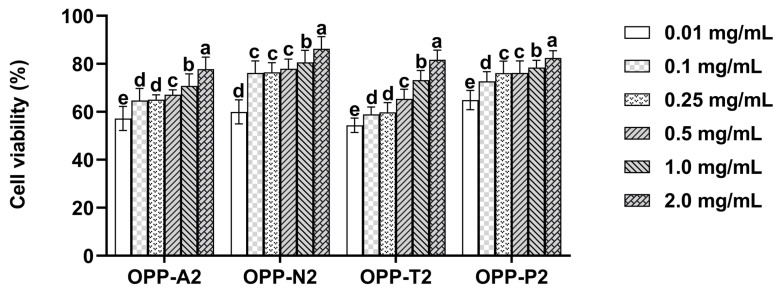
Protective effect of oyster peptides against H_2_O_2_-induced oxidative damage in LO2 cells. Values are expressed as means ± SD (*n* = 6). Different lowercase letters above bars indicate significant differences among concentrations within the same peptide fraction (*p* < 0.05).

Figure 3 illustrates the cytoprotective effects of oyster peptides against oxidative injury induced by H_2_O_2_ in LO2 cells. In all experimental groups, cell viability increased with rising peptide concentration, demonstrating a positive correlation between the protective efficacy and dosage. Significant improvements in cell viability were observed at both the lower (0.01–0.1 mg/mL) and higher (1–2 mg/mL) concentration ranges (*p* < 0.05). With respect to OPP-A1, OPP-A2, OPP-N2, and OPP-P2 groups, the protective effect reached a plateau within the concentration range of 0.1 and 0.5 mg/mL, indicating that the cytoprotective activity stabilized within this interval. To minimize potential interference with cellular metabolism at higher peptide levels, 0.5 mg/mL was selected for subsequent cellular assays.

### 3.6. Effect of Oyster Peptide Treatment on LDH Activity and MDA Content in LO2 Cells

**Figure 4:**
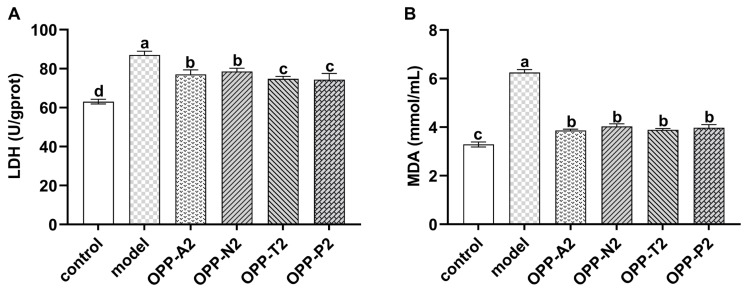
The effect of oyster peptides on LDH activity and MDA content in LO2 cells. (**A**) LDH activity, (**B**) MDA content. Values are expressed as means ± SD (*n* = 6). Different lowercase letters above bars indicate significant differences among different treatment groups (control, model, and peptide fractions) (*p* < 0.05).

LDH is a stable cytosolic enzyme released into the culture supernatant upon the loss of cell membrane integrity, with its activity positively correlated with the degree of membrane damage [26]. As shown in Figure 4A, the model group exhibited a markedly elevated LDH activity (87.05 U/gprot) compared with the control group (*p* < 0.05). Among them, OPP-T2 and OPP-P2 demonstrated the strongest protective effects, significantly outperforming OPP-A2 and OPP-N2 (*p* < 0.05). This result aligns with their previously observed superior capacity to inhibit lipid peroxidation and scavenge free radicals. Supporting this, Miyaji et al. [42] and Walrant et al. [43] suggested that trypsin tends to generate short peptide sequences with high membrane affinity, thereby stabilizing membrane structure. Similarly, Roque Borda et al. [44] showed that peptides (<10 kDa) obtained via papain digestion can integrate into the lipid bilayer of macrophage membranes, suppressing both the release of pro-inflammatory factors and the entry of extracellular Ca^2+^.

MDA is a terminal generated during lipid peroxidation and is widely used as a biomarker for oxidative damage [45]. As presented in Figure 4B, MDA content in the model group was significantly elevated compared with the control group (*p* < 0.05), confirming the successful establishment of the oxidative injury model. Treatment with oyster peptides significantly lowered intracellular MDA levels (*p* < 0.05), indicating effective mitigation of lipid peroxidation. No marked differences in MDA reduction were detected among the different peptide groups (*p* > 0.05). Collectively, these results suggest that the four low-molecular-weight oyster peptide fractions protected cellular and cell membrane stability, alleviated oxidative stress-induced lipid peroxidation, and significantly attenuated H_2_O_2_-induced injury in LO2 cells.

### 3.7. Effect of Oyster Peptide Treatment on SOD, GSH-Px and CAT Activities in LO2 Cells

**Figure 5:**
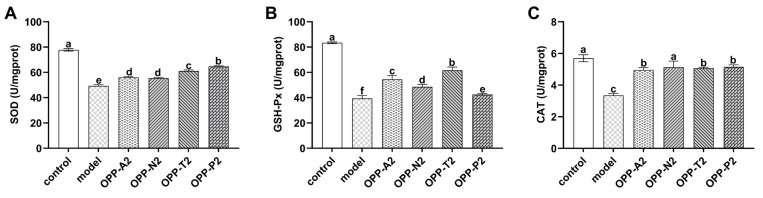
The effect of oyster peptides on oxidase activities in LO2 cells. (**A**) SOD activity, (**B**) GSH-Px activity, (**C**) CAT activity. Values are expressed as means ± SD (*n* = 6). Different lowercase letters above bars indicate significant differences among different treatment groups (control, model, and peptide fractions) (*p* < 0.05).

SOD, GSH-Px, and CAT are endogenous antioxidant enzymes that cooperatively scavenge ROS to maintain redox homeostasis. Elevations in their activities reflect the mitigation of oxidative damage [46,47]. As shown in Figure 5, compared to the model group, all four low-molecular-weight oyster peptide fractions significantly increased the activities of these three key antioxidant enzymes in LO2 cells (*p* < 0.05). Specifically, for SOD activity, OPP-T2 and OPP-P2 induced significantly greater enhancement than OPP-A2 and OPP-N2 (*p* < 0.05). OPP-T2 also produced the most pronounced increase in GSH-Px activity. OPP-N2 enhanced CAT activity more effectively than OPP-A2, OPP-T2, and OPP-P2 (*p* < 0.05).

These results suggest that all oyster peptide fractions may exert their cytoprotective effects by enhancing the activity of endogenous antioxidant enzymes. This aligns with the understanding that potent antioxidant peptides can function not only through direct radical scavenging but also by upregulating key cellular enzyme activities, as noted by Du et al. [48]. Notably, the hydrolysates from different proteases exhibited distinct profiles in enhancing specific enzyme activities. The trypsin-derived fraction OPP-T2 performed best overall, particularly in activating SOD and GSH-Px. In contrast, the papain-derived fraction OPP-P2 excelled specifically in boosting SOD activity, whereas the neutral protease-derived fraction OPP-N2 was most effective in elevating CAT activity. The alkaline protease-derived fraction OPP-A2 elicited a broad-spectrum and balanced enhancement, likely attributable to its wider cleavage specificity and resultant peptide diversity. These functional divergences underscore how protease cleavage specificity shapes the bioactivity of the resulting peptides. This observation is consistent with other reports. For instance, De Carvalho Cavenaghi et al. [49] found that the products obtained by hydrolyzing oyster mushrooms with three proteases (alcalase, flavourzyme and neutrase) showed significant differences in ABTS and DPPH radical scavenging rates and FRAP activity. Similarly, Wani et al. [1] identified pepsin-derived Porphyra protein hydrolysates as possessing the highest antioxidant potential among various enzymatic treatments.

## 4. Conclusions

This study systematically compared the hydrolysis effects of alkaline protease, neutral protease, trypsin, and papain on oyster protein and the antioxidant activities of their hydrolysates. Alkaline protease achieved the highest degree of hydrolysis, while papain showed the lowest. The low-molecular-weight fractions (OPP-A2, N2, T2, P2) played the primary role in in vitro radical scavenging and lipid peroxidation inhibition, indicating that molecular weight is a key determinant of antioxidant activity. The antioxidant activities of the low-molecular-weight fractions also exhibited significant differences. OPP-T2 and OPP-P2 showed superior in vitro antioxidant activity and enhanced cellular SOD activity compared to OPP-A2 and OPP-N2. OPP-T2 was the most effective in enhancing cellular GSH-Px activity.

This work provides a reference for the strategic selection of proteases to obtain oyster peptides with enhanced antioxidant properties. Although these findings identify promising candidates for further investigation, additional studies are required before practical application as functional foods. Future research should include endotoxin quantification to exclude contamination that may bias cellular responses, along with digestive stability tests, in vivo safety and efficacy assessment, and dose optimization. Structural characterization of the most active fractions is also recommended to elucidate their mechanisms of action.

## Figures and Tables

**Table 1:** Enzymatic hydrolysis conditions.

Protease	Substrate-to-Water Ratio (g/mL)	Enzyme Dosage (U/g)	pH	Temperature (°C)
Alkaline protease	1:2	4000	9.0	55
Neutral protease	1:2	3000	7.5	50
Trypsin	1:2	4000	8.0	50
Papain	1:3	4000	7.5	60

**Table 2:** The hydrolysis effects of different proteases on oyster protein.

Protease	Degree of Hydrolysis (%)	Molecular Weight Distribution of Hydrolysate (%)	
		>1 kDa	≤1 kDa
Alkaline protease	30.96 ± 0.59 ^a^	35.77	64.23
Neutral protease	24.40 ± 0.47 ^b^	46.30	53.70
Trypsin	25.07 ± 0.62 ^b^	69.34	30.66
Papain	18.29 ± 0.35 ^c^	45.05	54.95

Values are expressed as means ± SD (*n* = 3). Different lowercase superscript letters in the same column denote statistically significant differences (*p* < 0.05).

**Table 3:** The basic components and molecular weight distribution of the purified components of oyster peptides.

Protease	Fraction	Polysaccharide Content (%)	Protein Content (%)	Molecular Weight Distribution (%)	
				>1 kDa	≤1 kDa
Alkaline protease	OPP-A1	28.39 ±1.23 ^b^	56.30 ± 1.73 ^f^	80.97	19.03
	OPP-A2	5.27 ± 0.91 ^e^	79.90 ± 0.69 ^c^	24.53	75.47
Neutral protease	OPP-N1	26.78 ± 1.35 ^c^	58.72 ± 0.88 ^e^	75.66	24.34
	OPP-N2	7.83 ± 1.42 ^d^	84.91 ± 1.23 ^b^	0.00	100.00
Trypsin	OPP-T1	30.31 ± 0.88 ^a^	61.69 ± 1.16 ^d^	96.96	3.04
	OPP-T2	8.12 ± 1.19 ^d^	83.06 ± 0.71 ^b^	34.16	65.84
Papain	OPP-P1	25.33 ± 1.88 ^c^	57.60 ± 1.02 ^ef^	76.47	23.43
	OPP-P2	5.57 ± 1.26 ^e^	86.09 ± 0.94 ^a^	0.00	100.00

Values are expressed as means ± SD (*n* = 3). Different lowercase superscript letters within a column indicate significant differences (*p* < 0.05).

## Data Availability

The original contributions presented in this study are included in the article/Supplementary Materials. Further inquiries can be directed to the corresponding authors.

## Supplementary Materials

The following supporting information can be downloaded at: https://www.mdpi.com/article/10.3390/foods15061030/s1, Table S1: Alkaline protease response surface design and results; Table S2: Neutral protease response surface design and results; Table S3: Trypsin response surface design and results; Table S4: Papain response surface design and result; Table S5: Verification Experiment.

## References

[1] Wani H.M.U.D., Huang C.-Y., Singhania R.R., Patel A.K., Giri B.S., Chen C., Dong C.-D. (2024). Assessing and Optimizing the Bioactivities of Diverse Enzyme-Derived Protein Hydrolysates from Porphyra Yezoensis: Unlocking the Health Potential. J. Food Sci. Technol..

[2] Płóciniczak A., Bukowska-Olech E., Wysocka E. (2025). The Complexity of Oxidative Stress in Human Age-Related Diseases—A Review. Metabolites.

[3] Qin Y., Qian C., Li W., Wang Q., Sheng Q., Chen Z., Zhang W., Li W., Ge G., Yan Z. (2026). Oxidative Stress: Molecular Mechanisms, Diseases, and Therapeutic Targets. MedComm.

[4] Tu J., Peng J., Wen L., Li C., Xiao Z., Wu Y., Xu Z., Hu Y., Zhong Y., Miao Y. (2025). Antioxidant Peptides Derived from Woody Oil Resources: Mechanisms of Redox Protection and Emerging Therapeutic Opportunities. Pharmaceuticals.

[5] Xu B., Dong Q., Yu C., Chen H., Zhao Y., Zhang B., Yu P., Chen M. (2024). Advances in Research on the Activity Evaluation, Mechanism and Structure-Activity Relationships of Natural Antioxidant Peptides. Antioxidants.

[6] Nasri M. (2017). Protein Hydrolysates and Biopeptides. Advances in Food and Nutrition Research.

[7] Hu Y., Yang J., He C., Wei H., Wu G., Xiong H., Ma Y. (2022). Fractionation and Purification of Antioxidant Peptides from Abalone Viscera by a Combination of Sephadex G-15 and Toyopearl HW-40F Chromatography. Int. J. Food Sci. Tech..

[8] Liu J., Wu G., Yang J., He C., Xiong H., Ma Y. (2023). Abalone Visceral Peptides Containing Cys and Tyr Exhibit Strong in Vitro Antioxidant Activity and Cytoprotective Effects against Oxidative Damage. Food Chem. X.

[9] Liu B., Liu L., Li C., Guo T., Li C., Tian M., Fang T. (2025). The Alleviating Effect of Abalone Viscera Collagen Peptide in DSS-Induced Colitis Mice: Effect on Inflammatory Cytokines, Oxidative Stress, and Gut Microbiota. Nutrients.

[10] Zhang L., Li J., Zhou K. (2010). Chelating and Radical Scavenging Activities of Soy Protein Hydrolysates Prepared from Microbial Proteases and Their Effect on Meat Lipid Peroxidation. Bioresour. Technol..

[11] Mahmoud M., Mahmoud M. (2023). Promoting Soybean Protein Functional Properties by Enzymatic Hydrolysis: Characterization, Antioxidant Activity, Functional Properties and Application. Egypt. J. Chem..

[12] Alboofetileh M., Hamzeh A., Abdollahi M. (2021). Seaweed Proteins as a Source of Bioactive Peptides. Curr. Pharm. Des..

[13] Honrado A., Miguel M., Ardila P., Beltrán J.A., Calanche J.B. (2024). From Waste to Value: Fish Protein Hydrolysates as a Technological and Functional Ingredient in Human Nutrition. Foods.

[14] Zhang H., Huang L., Luo C., Li Z., Choong K., Cheong K.-L., Tan K. (2025). Contribution of China’s Bivalve Aquaculture to World’s Essential Amino Acid Production. npj Sci. Food.

[15] Chen H., Chen Y., Zheng H., Xiang X., Xu L. (2022). A Novel Angiotensin-I-Converting Enzyme Inhibitory Peptide from Oyster: Simulated Gastro-Intestinal Digestion, Molecular Docking, Inhibition Kinetics and Antihypertensive Effects in Rats. Front. Nutr..

[16] Chen T., Zhang F., Chen S., Zhao Y., Huang X., Huang F., Li C. (2024). Improvement Mechanism of Umami Peptides in Oyster Juice by Cooperative Enzymolysis of Alcalase and Trypsin Based on Peptidomics and Molecular Docking. J. Food Compos. Anal..

[17] Wu W., Wang X., Chen J., Tan J., Fu Y. (2025). Physicochemical and Flavor Characteristics of Maillard Reaction Products from Nile Tilapia Fish Skin Collagen Peptides Induced by Four Reducing Sugars. Foods.

[18] Ji H.-Y., Dai K.-Y., Liu C., Yu J., Jia X.-Y., Liu A.-J. (2022). Preparation, Antioxidant and Immunoregulatory Activities of a Macromolecular Glycoprotein from Salvia Miltiorrhiza. Foods.

[19] Jie Y., Zhao H., Zhang B. (2019). The Role of an Acidic Peptide in Controlling the Oxidation Process of Walnut Oil. Foods.

[20] Zheng Y., Nair S.K. (2023). YcaO-Mediated ATP-Dependent Peptidase Activity in Ribosomal Peptide Biosynthesis. Nat. Chem. Biol..

[21] Gan J., Ji Y., Sheng Q., Wang C., Shen X. (2025). Characterization of the Physicochemical Property, Antioxidant Activity and Hypoglycemic Potential of Quinoa Protein Hydrolysates. Food Biosci..

[22] Li Z., Xing Y., Liu P., Liao W., Miao L. (2025). Redox and Solvent-Stable Alkaline Serine Protease from Bacillus Patagoniensis DB-5: Heterologous Expression, Properties, and Biotechnological Applications. Front. Microbiol..

[23] Zhang Y., Yu S., Liu C., Jiang S., Wang H., Cheng Y., Guo Y., Qian H. (2025). Optimizing Enzymatic Processes for Enhanced Nutritional and Organoleptic Properties of Chicken Bones. Foods.

[24] Zaghloul E.H., Abuohashish H.M., El Sharkawy A.S., Abbas E.M., Ahmed M.M., Al-Rejaie S.S. (2023). Probiotic Potential of the Marine Isolate Enterococcus Faecium EA9 and in Vivo Evaluation of Its Antisepsis Action in Rats. Mar. Drugs.

[25] Wang J., Fang Z., Li Y., Sun L., Liu Y., Deng Q., Zhong S. (2022). Ameliorative Effects of Oyster Protein Hydrolysates on Cadmium-Induced Hepatic Injury in Mice. Mar. Drugs.

[26] Li T., Cheng X., Bao K., Wang L., Song M., Wang J., Wang S., Wang S., Wen T., Sun H. (2025). Structural Characterization, Antioxidant Activity, and Mechanism of Polysaccharides Isolated from Dictyophora Rubrovalvata Stipet. Int. J. Biol. Macromol..

[27] Burke M.C., Wang Y., Lee A.E., Dixon E.K., Castaneda C.A., Fushman D., Fenselau C. (2015). Unexpected Trypsin Cleavage at Ubiquitinated Lysines. Anal. Chem..

[28] Novinec M. (2017). Computational Investigation of Conformational Variability and Allostery in Cathepsin K and Other Related Peptidases. PLoS ONE.

[29] Terada K., Kurita T., Gimenez-Dejoz J., Masunaga H., Tsuchiya K., Numata K. (2022). Papain-Catalyzed, Sequence-Dependent Polymerization Yields Polypeptides Containing Periodic Histidine Residues. Macromolecules.

[30] Xu X., Zhao H., Yuan B., Yan S., Li Y. (2025). Effect of Different Enzyme Pretreatment on the Formation and Gel Properties of Soy Protein Nanofibrils. Food Biosci..

[31] GanjiVtan B., Hosseini Ghaboos S.H., Sadeghi Mahoonak A., Shahi T., Farzin N. (2025). Spray-dried Wheat Gluten Protein Hydrolysate Microcapsules: Physicochemical Properties, Retention of Antioxidant Capability, and Release Behavior under Simulated Gastrointestinal Digestion Conditions. Food Sci. Nutr..

[32] Sapatinha M., Camacho C., Pais-Costa A.J., Fernando A.L., Marques A., Pires C. (2024). Enzymatic Hydrolysis Systems Enhance the Efficiency and Biological Properties of Hydrolysates from Frozen Fish Processing Co-Products. Mar. Drugs.

[33] Zheng S.-L., Luo Q.-B., Suo S.-K., Zhao Y.-Q., Chi C.-F., Wang B. (2022). Preparation, Identification, Molecular Docking Study and Protective Function on HUVECs of Novel ACE Inhibitory Peptides from Protein Hydrolysate of Skipjack Tuna Muscle. Mar. Drugs.

[34] Li S., Li Y., Dou M., Zhang M., Zhao Z., Wu H., Zhu S., Obadina A.O. (2024). Glycogen and Zinc-Enriched Ferritin as Bioavailable Nanoparticulate Nutrients Released from Gastrointestinal Digestion of Pacific Oyster (Crassostrea Gigas). Food Chem..

[35] Fazhi X., Huihui P., Yang L., Lumu L., Kun Q., Xioling D. (2014). Separation and Purification of Small Peptides from Fermented Sesame Meal and Their Antioxidant Activities. Protein Pept. Lett..

[36] Tian S., Meng F., Du K. (2024). Physicochemical Properties and Structure Characteristics of Different Molecular Weight Peptides from Ultrasonic Assisted Papain Hydrolysate of Wheat Germ Albumin. Ind. Crops Prod..

[37] Majura J.J., Cao W., Chen Z., Htwe K.K., Li W., Du R., Zhang P., Zheng H., Gao J. (2022). The Current Research Status and Strategies Employed to Modify Food-Derived Bioactive Peptides. Front. Nutr..

[38] Afify A.E.-M.M.R., El Baroty G.S., El Baz F.K., Abd El Baky H.H., Murad S.A. (2018). Scenedesmus Obliquus: Antioxidant and Antiviral Activity of Proteins Hydrolyzed by Three Enzymes. J. Genet. Eng. Biotechnol..

[39] Kanwal R., Rehman A., Irfan M., Murtaza M.S., Tufail T., Murtaza M.A., Alsulami T., Khalifa I., Miao S. (2025). Investigating the Impact of Enzyme Diversity in Producing Edible Bird Nest Protein Hydrolysates: Techno-Functional, Structural, Molecular Docking, and in Vitro Digestibility. Food Struct..

[40] Goyal N., Kumar J., Hajare S.N., Gautam S. (2025). Cyclic Voltammetry for Antioxidant Assessment of Moth Bean Protein Hydrolysates: A Comparative Study with Spectrophotometric Methods. Food Meas..

[41] Rungchang S., Sringarm C., Numthuam S., Tosuk N., Thongsuk T., Phinyo M., Winuprasith T., Jiamyangyuen S. (2025). Hydrolysis Kinetics Amino Acid Profiling and Antioxidant Properties of Enzymatic Hydrolysates from Desalted Egg White. Sci. Rep..

[42] Miyaji A., Satou K., Baba T. (2020). Influence of Tryptic Hydrolysis on the Enzymatic Function of the Membrane-Bound Form of Particulate Methane Monooxygenase from Methylosinus Trichosporium OB3b. J. Biotechnol..

[43] Walrant A., Cardon S., Burlina F., Sagan S. (2017). Membrane Crossing and Membranotropic Activity of Cell-Penetrating Peptides: Dangerous Liaisons?. Acc. Chem. Res..

[44] Roque-Borda C.A., Primo L.M.D.G., Medina-Alarcón K.P., Campos I.C., Nascimento C.D.F., Saraiva M.M.S., Berchieri Junior A., Fusco-Almeida A.M., Mendes-Giannini M.J.S., Perdigão J. (2025). Antimicrobial Peptides: A Promising Alternative to Conventional Antimicrobials for Combating Polymicrobial Biofilms. Adv. Sci..

[45] Xu Z., Hu Q., Xie M., Liu J., Su A., Xu H., Yang W. (2023). Protective Effects of Peptide KSPLY Derived from Hericium Erinaceus on H2O2-Induced Oxidative Damage in HepG2 Cells. Food Sci. Hum. Wellness.

[46] Zhang A., Cui L., Tu X., Liang Y., Wang L., Sun Y., Kang X., Wu Z. (2024). Peptides Derived from Casein Hydrolyzed by Lactobacillus: Screening and Antioxidant Properties in H2O2-Induced HepG2 Cells Model. J. Funct. Foods.

[47] Liu W., Ren J., Wu H., Zhang X., Han L., Gu R. (2023). Inhibitory Effects and Action Mechanism of Five Antioxidant Peptides Derived from Wheat Gluten on Cells Oxidative Stress Injury. Food Biosci..

[48] Du B., Zhang C., Deng G., Zhang S., Wang S., Guan Y., Huang Y. (2024). Identification of Novel Antioxidant Collagen Peptides for Preventing and Treating H2O2-Induced Oxidative Stress in HepG2 Cells through in Vitro and in Silico Approaches. J. Sci. Food Agric..

[49] De Carvalho Cavenaghi D.F.L., De Barros W.M., De Castro R.J.S. (2025). Protein Hydrolysates from Pleurotus Spp. Mushrooms as a Source of Antioxidant Peptides and Their Stability after Gastrointestinal Digestion. Food Humanit..

