# Supplementary material for: Protease Selection Influences Molecular Weight, In Vitro Antioxidant Activity and LO2 Cellular Protective Effects of Oyster Protein Hydrolysates
Source: Foods. 2026 Mar 16;15(6):1030. doi: 10.3390/foods15061030 (PMC13024773; doi:10.3390/foods15061030)
Supplement: Supplementary file 1 [file foods-15-01030-s001.zip › foods-4182099-supplementary.pdf]

**Table S1.** Alkaline protease response surface design and results

| Run | A: pH | B: Temperature (°C) | C: Substrate-to-water ratio (g/mL) | D: Enzyme dosage (U/g) | R1: Hydrolysis degree (%) |
|---|---|---|---|---|---|
| 1 | 9.5 | 55 | 1:2 | 2000 | 23.06±0.32 |
| 2 | 9.5 | 50 | 1:2 | 3000 | 22.56±0.28 |
| 3 | 10.0 | 50 | 1:2 | 4000 | 23.20±0.72 |
| 4 | 9.5 | 50 | 1:3 | 2000 | 17.19±0.64 |
| 5 | 9.5 | 45 | 1:3 | 3000 | 21.82±0.57 |
| 6 | 9.5 | 50 | 1:2 | 3000 | 20.82±0.41 |
| 7 | 9.0 | 50 | 1:2 | 4000 | 30.86±0.62 |
| 8 | 10.0 | 50 | 1:2 | 2000 | 25.14±0.77 |
| 9 | 9.0 | 50 | 1:2 | 2000 | 25.23±0.16 |
| 10 | 9.5 | 50 | 1:1 | 2000 | 22.36±0.54 |
| 11 | 10.0 | 50 | 1:3 | 3000 | 19.83±0.43 |
| 12 | 10.0 | 45 | 1:2 | 3000 | 23.53±0.58 |
| 13 | 10.0 | 50 | 1:1 | 3000 | 23.18±0.68 |
| 14 | 9.5 | 45 | 1:2 | 4000 | 23.76±0.82 |
| 15 | 9.5 | 55 | 1:3 | 3000 | 18.15±0.32 |
| 16 | 9.5 | 50 | 1:3 | 4000 | 22.06±0.51 |
| 17 | 9.0 | 45 | 1:2 | 3000 | 24.75±0.46 |
| 18 | 9.0 | 50 | 1:1 | 3000 | 22.51±0.18 |
| 19 | 9.5 | 45 | 1:2 | 2000 | 22.48±0.32 |
| 20 | 9.0 | 50 | 1:3 | 3000 | 25.03±0.59 |
| 21 | 9.5 | 50 | 1:2 | 3000 | 20.82±0.22 |
| 22 | 9.5 | 45 | 1:1 | 3000 | 19.08±0.49 |
| 23 | 10.0 | 55 | 1:2 | 3000 | 20.84±0.32 |
| 24 | 9.5 | 50 | 1:1 | 4000 | 23.39±0.55 |
| 25 | 9.5 | 55 | 1:1 | 3000 | 22.69±0.73 |
| 26 | 9.5 | 55 | 1:2 | 4000 | 25.03±0.24 |
| 27 | 9.0 | 55 | 1:2 | 3000 | 26.39±0.62 |

**Table S2.** Neutral protease response surface design and results

| Run | A:pH | B: Temperature (°C) | C: Substrate-to-water ratio (g/mL) | D: Enzyme dosage (U/g) | R1: Hydrolysis degree (%) |
|---|---|---|---|---|---|
| 1 | 7.5 | 45 | 1:2 | 2000 | 17.37±0.57 |
| 2 | 8.0 | 50 | 1:2 | 2000 | 14.67±0.41 |
| 3 | 7.0 | 45 | 1:2 | 3000 | 16.96±0.26 |

| | | | | | |
|---|---|---|---|---|---|
| 4 | 8.0 | 55 | 1:2 | 3000 | 26.62±0.23 |
| 5 | 8.0 | 45 | 1:2 | 3000 | 18.58±0.38 |
| 6 | 7.5 | 50 | 1:1 | 4000 | 18.02±0.66 |
| 7 | 7.5 | 55 | 1:1 | 3000 | 19.34±0.39 |
| 8 | 7.5 | 45 | 1:2 | 4000 | 18.84±0.42 |
| 9 | 8.0 | 50 | 1:3 | 3000 | 18.84±0.65 |
| 10 | 7.5 | 55 | 1:2 | 4000 | 17.36±0.31 |
| 11 | 7.5 | 50 | 1:1 | 2000 | 17.74±0.52 |
| 12 | 7.5 | 50 | 1:2 | 3000 | 24.80±0.37 |
| 13 | 7.0 | 50 | 1:2 | 4000 | 15.14±0.32 |
| 14 | 7.0 | 50 | 1:3 | 3000 | 19.37±0.56 |
| 15 | 7.5 | 50 | 1:3 | 2000 | 18.15±0.73 |
| 16 | 7.5 | 45 | 1:3 | 3000 | 18.32±0.68 |
| 17 | 8.0 | 50 | 1:1 | 3000 | 18.90±0.81 |
| 18 | 7.0 | 50 | 1:2 | 2000 | 19.91±0.12 |
| 19 | 7.5 | 45 | 1:1 | 3000 | 19.27±0.18 |
| 20 | 7.0 | 55 | 1:2 | 3000 | 20.03±0.24 |
| 21 | 7.5 | 55 | 1:3 | 3000 | 26.78±0.39 |
| 22 | 7.0 | 50 | 1:1 | 3000 | 20.28±0.51 |
| 23 | 7.5 | 55 | 1:2 | 2000 | 24.84±0.43 |
| 24 | 7.5 | 50 | 1:2 | 3000 | 26.45±0.62 |
| 25 | 7.5 | 50 | 1:2 | 3000 | 24.80±0.57 |
| 26 | 8.0 | 50 | 1:2 | 4000 | 18.69±0.33 |
| 27 | 7.5 | 50 | 1:3 | 4000 | 17.74±0.67 |

**Table S3.** Trypsin response surface design and results

| Run | A: pH | B: Temperature (°C) | C: Substrate-to-water ratio (g/mL) | D: Enzyme dosage (U/g) | R1: Hydrolysis degree (%) |
|---|---|---|---|---|---|
| 1 | 7.5 | 50 | 1:1 | 3000 | 18.30±0.51 |
| 2 | 7.5 | 50 | 1:2 | 2000 | 24.95±0.62 |
| 3 | 8.0 | 55 | 1:2 | 2000 | 17.72±0.14 |
| 4 | 7.5 | 45 | 1:2 | 3000 | 11.84±0.37 |
| 5 | 8.0 | 50 | 1:3 | 2000 | 22.86±0.35 |
| 6 | 8.0 | 50 | 1:2 | 3000 | 24.12±0.66 |
| 7 | 7.5 | 50 | 1:2 | 4000 | 23.97±0.71 |
| 8 | 8.5 | 45 | 1:2 | 3000 | 12.58±0.39 |
| 9 | 8.5 | 50 | 1:1 | 3000 | 16.93±0.42 |
| 10 | 8.0 | 45 | 1:3 | 3000 | 12.60±0.53 |

| | | | | | |
|---|---|---|---|---|---|
| 11 | 8.0 | 45 | 1:1 | 3000 | 11.81±0.12 |
| 12 | 8.0 | 45 | 1:2 | 2000 | 13.05±0.23 |
| 13 | 8.5 | 50 | 1:3 | 3000 | 21.12±0.37 |
| 14 | 8.0 | 50 | 1:2 | 3000 | 23.37±0.45 |
| 15 | 8.0 | 50 | 1:1 | 4000 | 23.57±0.24 |
| 16 | 8.0 | 55 | 1:2 | 4000 | 19.30±0.51 |
| 17 | 7.5 | 50 | 1:3 | 3000 | 15.96±0.22 |
| 18 | 8.0 | 50 | 1:1 | 2000 | 18.92±0.31 |
| 19 | 8.5 | 50 | 1:2 | 4000 | 24.30±0.53 |
| 20 | 8.0 | 50 | 1:3 | 4000 | 24.23±0.61 |
| 21 | 8.0 | 50 | 1:2 | 3000 | 22.96±0.46 |
| 22 | 7.5 | 55 | 1:2 | 3000 | 18.92±0.28 |
| 23 | 8.5 | 50 | 1:2 | 2000 | 20.52±0.29 |
| 24 | 8.0 | 45 | 1:2 | 4000 | 12.45±0.35 |
| 25 | 8.0 | 55 | 1:3 | 3000 | 13.96±0.42 |
| 26 | 8.5 | 55 | 1:2 | 3000 | 17.39±0.26 |
| 27 | 8.0 | 55 | 1:1 | 3000 | 14.54±0.18 |

**Table S4.** Papain response surface design and result

| Run | A: pH | B: Temperature (°C) | C: Substrate-to-water ratio (g/mL) | D: Enzyme dosage (U/g) | R1: Hydrolysis degree (%) |
|---|---|---|---|---|---|
| 1 | 6.5 | 55 | 1:2 | 2000 | 13.54±0.53 |
| 2 | 7.0 | 60 | 1:2 | 2000 | 9.42±0.35 |
| 3 | 6.5 | 60 | 1:2 | 3000 | 13.97±0.47 |
| 4 | 7.0 | 50 | 1:3 | 3000 | 12.24±0.15 |
| 5 | 6.5 | 55 | 1:3 | 3000 | 16.40±0.24 |
| 6 | 7.0 | 55 | 1:1 | 4000 | 14.12±0.38 |
| 7 | 6.5 | 55 | 1:2 | 4000 | 15.53±0.72 |
| 8 | 7.5 | 55 | 1:2 | 4000 | 17.04±0.61 |
| 9 | 7.0 | 50 | 1:2 | 2000 | 9.72±0.54 |
| 10 | 7.0 | 60 | 1:1 | 3000 | 9.95±0.41 |
| 11 | 6.5 | 50 | 1:2 | 3000 | 10.64±0.37 |
| 12 | 7.0 | 55 | 1:1 | 2000 | 9.45±0.45 |
| 13 | 7.0 | 55 | 1:3 | 2000 | 12.48±0.29 |
| 14 | 7.0 | 55 | 1:2 | 3000 | 12.55±0.21 |
| 15 | 7.5 | 60 | 1:2 | 3000 | 13.30±0.52 |
| 16 | 7.5 | 55 | 1:2 | 2000 | 11.50±0.17 |
| 17 | 7.0 | 60 | 1:3 | 3000 | 16.67±0.11 |
| 18 | 7.5 | 50 | 1:2 | 3000 | 13.41±0.63 |

| | | | | | |
|---|---|---|---|---|---|
| 19 | 7.0 | 50 | 1:2 | 4000 | 16.44±0.51 |
| 20 | 7.0 | 55 | 1:3 | 4000 | 15.46±0.38 |
| 21 | 7.0 | 60 | 1:2 | 4000 | 15.44±0.32 |
| 22 | 7.0 | 55 | 1:2 | 3000 | 12.87±0.46 |
| 23 | 7.0 | 50 | 1:1 | 3000 | 10.15±0.31 |
| 24 | 7.5 | 55 | 1:1 | 3000 | 12.99±0.19 |
| 25 | 7.0 | 55 | 1:2 | 3000 | 12.13±0.33 |
| 26 | 7.5 | 55 | 1:3 | 3000 | 14.99±0.56 |
| 27 | 6.5 | 55 | 1:1 | 3000 | 14.68±0.41 |

**Table S5.** Verification Experiment

| Alkaline protease | | | | | |
|---|---|---|---|---|---|
| Model | pH (A) | Tempe-rature (B) | Substrate-to-water ratio (C) | Enzyme dosage (Da) | Hydrolysis degree (%) |
| Simulated prediction | 9.0 | 54.40 | 1.00:1.88 | 3999 | 31.68 |
| Actual experiment | 9.0 | 55.00 | 1.00:2.00 | 4000 | 30.96±0.59 |
| Neutral protease | | | | | |
| Simulated prediction | 7.4 | 51.30 | 1.00:1.99 | 2782 | 24.98 |
| Actual experiment | 7.5 | 50.00 | 1.00:2.00 | 3000 | 24.43±0.47 |
| Trypsin | | | | | |
| Simulated prediction | 8.2 | 50.84 | 1.00:2.07 | 4000 | 25.68 |
| Actual experiment | 8.0 | 50.00 | 1.00:2.00 | 4000 | 25.07±0.62 |
| Papain | | | | | |
| Simulated prediction | 7.5 | 57.84 | 1.00:3.00 | 4000 | 19.04 |
| Actual experiment | 7.5 | 60.00 | 1.00:3.00 | 4000 | 18.29±0.35 |
